# Occurrence and Distribution of Microcystins in Lake Taihu, China

**DOI:** 10.1155/2013/838176

**Published:** 2013-06-16

**Authors:** Hiroshi Sakai, Aimin Hao, Yasushi Iseri, Song Wang, Takahiro Kuba, Zhenjia Zhang, Hiroyuki Katayama

**Affiliations:** ^1^Department of Urban Engineering, The University of Tokyo, 7-3-1 Hongo, Bunkyo-ku, Tokyo 113-8656, Japan; ^2^Department of Urban and Environmental Engineering, Kyushu University, 744 Motooka, Nishi-ku, Fukuoka 819-0395, Japan; ^3^West Japan Engineering Consultants, Inc., 1-1-1 Watanabe-dori, Chuo-ku, Fukuoka 810-0004, Japan; ^4^School of Environmental Science and Engineering, Shanghai Jiao Tong University, 800 Dongchuan Road, Shanghai 200240, China

## Abstract

The occurrence and distribution of microcystins were investigated in Lake Taihu, the third largest lake in China. An extensive survey, larger and broader in scale than previous studies, was conducted in summer 2010. The highest microcystin concentration was found at southern part of Taihu, which was newly included in this survey. In northern coastal areas, total cellular concentrations of 20 to 44 μg/L were observed. In northern offshore waters, levels were up to 4.8 μg/L. Microcystin occurrence was highly correlated with chemical oxygen demand, turbidity, and chlorophyll-a. Extracellular/total cellular microcystin (E/T) ratios were calculated and compared to other water quality parameters. A higher correlation was found using E/T ratios than original microcystin values. These results show that algal blooms are having a severe impact on Lake Taihu, and further and extensive monitoring and research are required to suppress blooms effectively.

## 1. Introduction

The presence of microcystins in water systems is a major threat to human health. Microcystins are contained in *Microcystis* cells and are released when these cells die. The toxicity of microcystin-LR is severe, with an LD_50_ value of about 50 *μ*g/kg for mice, which is lower than that of potassium cyanide [1]. *Microcystis* blooms occur in lakes and reservoirs, causing problems for both drinking water supplies and recreational water use. The World Health Organization recommends that microcystin values not exceed 1 *μ*g/L in drinking water [2] and 20 *μ*g/L in recreational water [3]. The largest microcystin outbreak occurred in 1996 in Brazil, where microcystin-contaminated tap water was supplied to a hemodialysis center, resulting in the deaths of 88 patients [4, 5]. Many other outbreaks of microcystins and other cyanobacterial toxins have occurred; these outbreaks did not result in human deaths, but cases of diarrhea, nausea, and vomiting were reported [6–14]. One study indicated that microcystins may promote liver tumors [15]. 

Lake Taihu is the third largest freshwater lake in China and recently has been suffering from severe algal blooms [16]. The lake has a surface area of 2,428 km^2^ [17], and its waters have been utilized for drinking water, sewage disposal, fisheries, and aquaculture [18]. Before 1980, the lake experienced fewer algal blooms, and its water quality met standards for drinking water. However, due to the rapid development of the economy and the intensive use of the lake, water pollution has become increasingly serious in Lake Taihu since the 1980s [17]. In 2006, 93% of the lake was in a state of mid-level eutrophication, versus only 6.1% in 1997. Therefore, eutrophication, cyanobacterial blooms, and the occurrence of cyanotoxins are serious problems in the region.

To examine the eutrophication problem, a three-dimensional model was established to simulate the occurrence of algae in Lake Taihu [19]. This model incorporated the effects of weather, especially wind speed and direction, and succeeded, to a certain extent, in simulating the eutrophication conditions in the lake. At Lake Taihu, southeast trade winds prevail, and this wind could be a primary factor driving circulation in the lake [19]. Various studies have confirmed the presence of microcystins in Lake Taihu [18, 20–25]. Previous studies recorded levels of around 20 *μ*g/L total cellular microcystin in Meiliang Bay in 2001 [22] and 30 *μ*g/L in Gonghu Bay in 2008 [25]. These bays experienced a peak microcystin concentration in August [18, 20, 21] or October [20, 22, 25]. The aforementioned studies also determined algal species [21, 24] and relationships with other water quality parameters [23, 25]. However, the only previous studies on microcystin occurrence focused only on Meiliang or Gonghu bay. Moreover, only a couple of sampling points was surveyed in those areas, particularly in coastal areas. As already revealed by previous research [17, 19], eutrophication occurs all over Lake Taihu not only in Meiliang and Gonghu bays. Therefore, microcystin occurrence should be investigated over the whole area of Lake Taihu, as well as in those two bays. 

Although growth information is important for the formulation of countermeasures, algal growth stage has not been examined carefully in previous studies. The growth stage of a bloom will affect remediation strategies; in the early growth phase, nutrient elimination is useful for suppressing further blooming, while in the death phase physical removal of the bloom is recommended. In the present study, to help offset the lack of information on growth stage from the existing parameters, the extracellular/total cellular microcystin (E/T) ratio was used. Microcystins are always contained in algal cells and are released into the environment only when those cells die [26, 27]. There are fewer dead cells in the growth phase, leading to lower extracellular microcystin concentrations and a lower E/T ratio. In contrast, there are more dead cells in the death phase, leading to higher extracellular microcystin concentrations and greater E/T ratios. The viability of the E/T ratio was investigated as another objective of this research. Based on our extensive survey results, we suggest further research directions for managing algal blooms in Lake Taihu. 

## 2. Materials and Methods

### 2.1. Study Area and Sampling Locations

**Figure 1:**
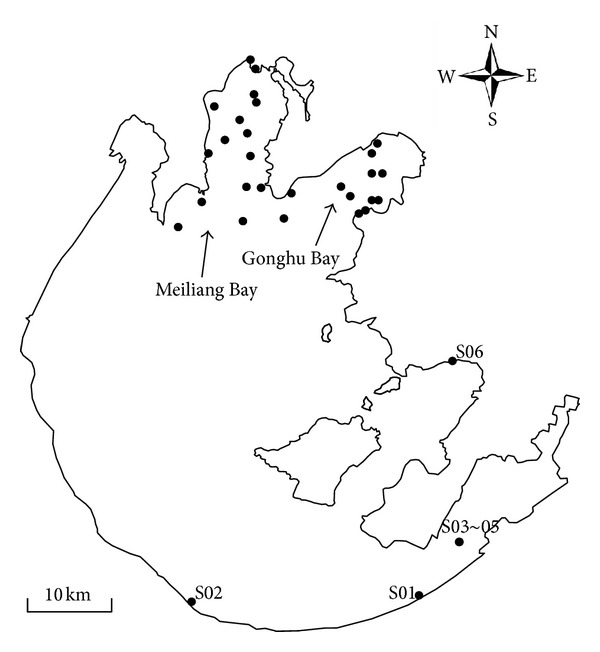
Map showing sampling locations on Lake Taihu.

Lake Taihu is located in Jiangsu and Zhejiang Provinces, China. It is shallow (mean depth 1.9 m) and has a large surface area (2,428 km^2^). It serves as an important resource for drinking water, irrigation, aquaculture, and industrial water, in addition to being a popular recreational and tourist attraction [25]. Samples were collected from July 30 to August 2, 2010, from 33 locations across the lake, ranging from Meiliang Bay and Gonghu Bay to the south and eastern parts of the lake (Figure 1). Among the 33 locations, 10 sites (G01–G10) were located in Gonghu Bay, 17 (M01–M17) were located around Meiliang Bay and in the center of the lake, and the remaining 6 sites (S01–S06) were located in eastern and southern parts of the lake. Sampling was conducted in offshore as well as coastal areas.

### 2.2. Analysis

Water temperature, dissolved oxygen (DO), turbidity, chlorophyll, pH, oxidation-reduction potential (ORP), and electrical conductivity were measured at the time of sampling using a multiprobe (Datasonde 5X, HACH). Chemical oxygen demand (COD Cr) was measured using an HACH DR2800 colorimeter. Dissolved organic carbon (DOC), nitrate, and phosphate were also measured after 0.45 *μ*m filtration. DOC was measured using a TOC-V (Shimadzu), and nitrate and phosphate were measured using an HACH DR2800 colorimeter. Intracellular and extracellular microcystins were measured using ELISA, as described in previous reports [26, 28]. The detection limit of this method is 50 ng/L as a microcystin-LR equivalent. 

## 3. Results

### 3.1. Overall Occurrence of Microcystins

**Figure 2:**
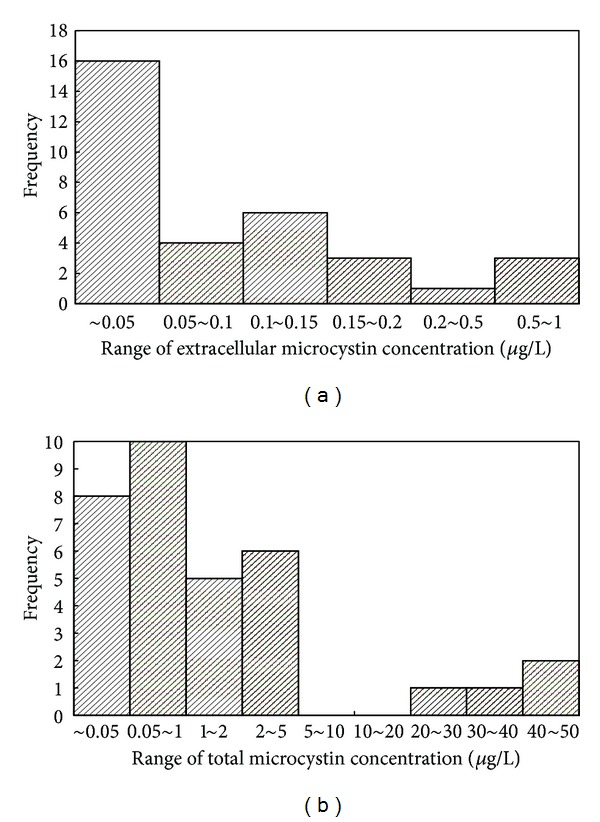
Frequency distributions of total cellular and extracellular microcystins.

Microcystins were detected in 25 of 34 samples measured, showing a broad occurrence across Lake Taihu. Microcystin concentrations were expressed as total cellular microcystin and extracellular microcystin; the frequency distributions are shown in Figures 2(a) and 2(b). Detailed data are shown in the Supporting Material (See Table S1 available online at http://dx.doi.org/10.1155/2013/838176).

The highest recorded extracellular microcystin concentration was close to 1 *μ*g/L, the drinking water guideline value suggested by the WHO. The highest concentration was 0.96 *μ*g/L at M01 (Meiliang Bay) on July 30, followed by 0.88 *μ*g/L at M06 (also Meiliang Bay) on August 2 and 0.79 *μ*g/L at S02 (southern Taihu) on July 29. For other samples, the highest recorded extracellular microcystin concentration was 0.22 *μ*g/L. There were three samples in the range of 0.15–0.2 *μ*g/L, six samples between 0.1 and 0.15 *μ*g/L and four samples between 0.05 and 0.1 *μ*g/L.

Total cellular microcystin concentration was higher than 1 *μ*g/L for 15 of the 34 samples, with 4 samples exceeding the WHO recommended 20 *μ*g/L limit for water for recreational use. The highest concentration was 50 *μ*g/L in sample S02 from Huzhou city, in the southern part of the lake. Levels were also high in the samples from most north shores of Meiliang Bay, at 44 *μ*g/L at M01 on July 30 and 35 *μ*g/L at M06 on August 2. A concentration of 20 *μ*g/L was observed on July 31 at sampling point M02 on the west shore of Meiliang Bay. Another 11 samples from Gonghu Bay and Meiliang Bay also exceeded 1 *μ*g/L. As this is the concentration of total cellular microcystin in samples, it is not an immediate threat to human health. However, serious impacts on human health would occur if these microcystins were to be released into waters.

### 3.2. Spatial Distribution of Microcystins in Gonghu Bay

**Figure 3:**
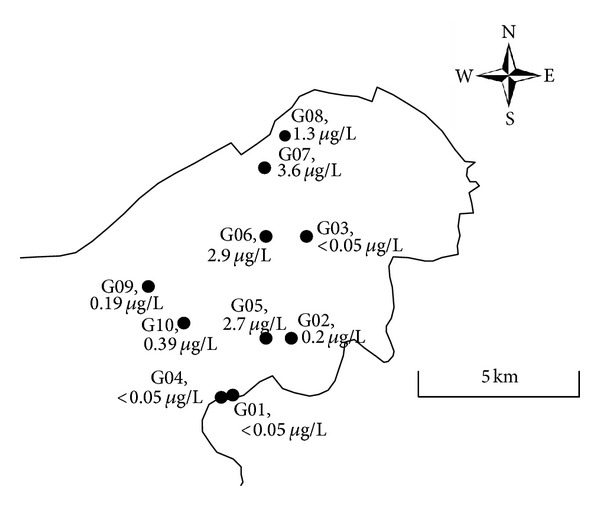
Microcystin distributions in Gonghu Bay.

In Gonghu Bay, 10 samples were taken and analyzed for various water quality parameters including microcystins. Total cellular microcystin concentrations and sampling point locations are shown in Figure 3. Two sampling series were conducted, on July 30 and August 1, in a north-south direction. Three samples (G01–G03) were taken on July 30, and five samples (G04–G08) were taken on August 1. The two other samples (G09 and G10) were taken on August 2, from the west part of Gonghu Bay. Results from the west and central parts of Gonghu Bay were compared. On July 30, the total cellular microcystin concentration was 0.2 *μ*g/L at sampling point G02 and was under the detection limit of 0.05 *μ*g/L at the other two points. The weather on July 30 was cloudy and stormy.

On August 1, microcystin concentrations were under 0.05 *μ*g/L at the shore (G04) and ranged from 1.4 to 3.6 *μ*g/L offshore (G05–G08). Although the locations of some sampling points were close to each other, some differences were observed between concentrations on July 30 and August 1. Total cellular microcystin concentration ranged from 0.2 *μ*g/L on July 30 to more than 1.4 *μ*g/L on August 1 offshore. This difference could be attributed to changing weather conditions. On July 30, it was cloudy, and a rainstorm began during sampling. This disturbed the surface of the lake and dispersed algae, which would have led to lower recorded concentrations of microcystin on July 30. Conversely, the weather was calm and sunny on August 1. Samples were taken on August 2 in the west part of Gonghu Bay. Concentrations were 0.39 *μ*g/L at sampling point G10 and 0.19 *μ*g/L at sampling point G09. Clear differences were found among sampling locations and dates, possibly due to weather conditions. 

### 3.3. Spatial Distribution of Microcystins in Meiliang Bay

**Figure 4:**
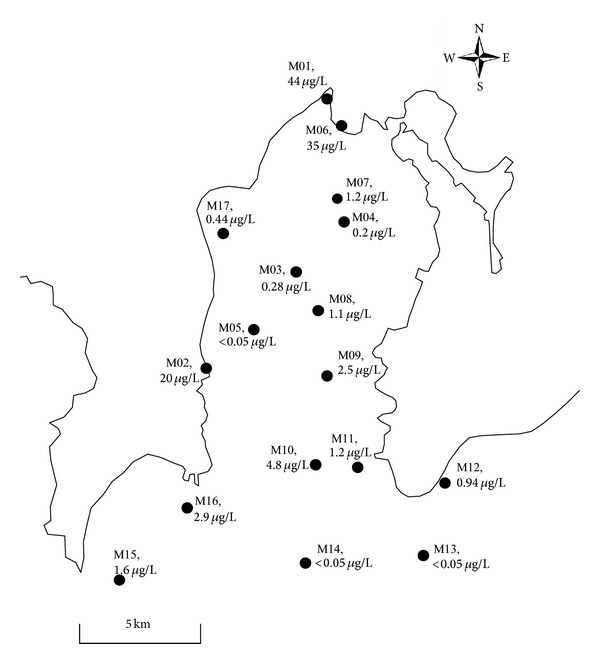
Microcystin distributions in Meiliang Bay.

In Meiliang Bay, 17 samples were taken, and total cellular microcystin concentrations were compared as shown in Figure 4. Higher concentrations were observed on the north and west shores of the bay. Concentrations were 44 *μ*g/L at sampling point M01 and 35 *μ*g/L at M06 on the north shore and 20 *μ*g/L at M02 on the west shore. Among the other 14 samples taken from offshore waters, 3 had concentrations higher than 2 *μ*g/L, with the highest being 4.8 *μ*g/L at M10 in the middle of the mouth of Meiliang Bay. The second highest concentration was 2.9 *μ*g/L at M16 in the western section of the mouth of the bay, and the third highest one was 2.5 *μ*g/L at M09, north of the M10 sampling point. These three points seem to form a belt that crosses Meiliang Bay from the northeast to the southwest. Four samples (M07, M08, M11, and M16) had concentrations ranging between 1 *μ*g/L and 2 *μ*g/L. Sampling points located around the belt area included M09, M10, and M16. In other areas, microcystin concentrations were lower than 1 *μ*g/l. At M13 and M14, which were closer to the center of the lake, concentrations were under the detection limit of 50 ng/L.

Great variation in the concentration was found even within Meiliang Bay, with 20–44 *μ*g/L observed in coastal areas and 1∼5 *μ*g/L observed around the mouth of the bay, forming a belt from a northeast to a southwest direction. This direction is perpendicular to the prevailing wind direction at Lake Taihu [19]. In other areas, total cellular microcystin concentrations were lower than 1 *μ*g/L. 

### 3.4. Microcystin Occurrence in South and East Taihu

In the southern and eastern regions of Lake Taihu, one sample was taken at Suzhou city, three samples were taken in eastern Taihu, and one sample was taken at Huzhou city; total cellular microcystin concentrations were 0.40 *μ*g/L, 0.05–0.30 *μ*g/L, and 50 *μ*g/L, respectively. Extracellular microcystin concentrations were 0.79 *μ*g/L at Huzhou city and less than 0.05 *μ*g/L in other samples. Although there was only one microcystin “hot-spot,” this location, at Huzhou city was the most severe in all samples. Therefore, broader investigations should be conducted to better understand the overall occurrence of microcystins in Lake Taihu.

### 3.5. Relationships between Microcystins and Other Water Quality Parameters

Relationships between total cellular and intracellular microcystins and other water quality parameters are summarized in Table 1. The relationships between microcystins and physical parameters (pH, ORP, and EC) were relatively weak, their correlation coefficients ranging from −0.20 to 0.18. Higher correlation coefficients were found between both extracellular and total cellular microcystins and turbidity, chlorophyll-a, and COD: 0.96 and 0.98 for turbidity, 0.71 and 0.61 for chlorophyll-a, and 0.75 and 0.73 for COD, respectively. The correlations between DO and microcystins were not so high at 0.28 and 0.18 for extracellular and total cellular microcystin, respectively. The higher correlations with COD and chlorophyll-a could be attributed to algal biomass, as reported in previous research [23, 25]. The higher correlation with chlorophyll-a and lower correlation with DO suggest that live algal species and dead algal scum coexisted in the samples, which otherwise would have shown high coefficients with DO as well as with chlorophyll-a. Correlation coefficients with DOC, NO_3_-N, and PO_4_-P were also low, at 0.37 and 0.32 for DOC, 0.01 and 0.15 for NO_3_-N, and 0.24 and 0.17 for PO_4_-P for extracellular and total cellular microcystins, respectively. As algae photosynthesize by utilizing atmospheric carbon dioxide, it could be expected that DOC and microcystins would be independent. The correlation coefficients of NO_3_-N and PO_4_-P with microcystins were lower than that with DOC. This suggests that there was no relationship between nutrients and extracellular or total cellular microcystins. Moreover, it can be speculated that other factors, such as meteorological factors (e.g., wind), contributed to the occurrence and distribution of microcystins in Lake Taihu.

### 3.6. Extracellular/Total Cellular Microcystin Ratios

E/T ratios, which characterize an algal bloom by its growth phase, were calculated (Table 2). Our previous study showed E/T ratios of 8.3∼15.0% during the exponential growth phase of a pure culture of *M. aeruginosa* PCC 7806 [28]. Thus, this ratio was employed to characterize the algal bloom.

In the present study, G02 had an E/T ratio of 107%, while other samples had smaller E/T ratios. The high ratio at G02 may have been due to the death phase of the bloom. G02 was sampled during stormy weather, and algal cells may have been killed off by the storm, thus releasing intracellular microcystins. In contrast, the other samples may have been in the growth phase. In reference to the E/T ratios of pure culture [28], samples G8 to M17 could be considered to be in the normal growth phase. Samples with values lower than 8.3% might have been affected by external factors.

The correlations of E/T ratios with other water quality parameters were investigated (Table 3). E/T ratios were correlated with chlorophyll-a, pH, DO, NO_3_-N, and PO_4_-P, with correlation coefficients of 0.27, 0.31, 0.30, −0.40, and 0.27, respectively. The relatively high correlations with DO, pH, and nutrient parameters should be noted, as they are higher than their correlation coefficients with microcystins, as shown in Table 1. Therefore, E/T ratios, rather than conventional parameters, could be used to express bloom growth conditions. The positive correlation coefficients with chlorophyll-a, DO, pH, and PO_4_-P mean that higher E/T ratios were correlated with more algal growth in Lake Taihu. The negative correlation coefficient with NO_3_-N suggests that phosphate concentration could be a limiting factor for algal growth in Lake Taihu. These results indicate that E/T ratios would be more suitable than microcystin concentrations for evaluating bloom growth conditions.

## 4. Discussion

### 4.1. Occurrence and Distribution of Microcystins in Lake Taihu

In this study, we investigated the occurrence and distribution of microcystins in Lake Taihu. First, we investigated various locations of the lake and revealed the occurrence of microcystins across a broad area. Previous studies only considered microcystin occurrence in Meiliang and Gonghu bays and showed total cellular microcystin concentrations of around 20 *μ*g/L in Meiliang Bay in 2001 [22] and 30 *μ*g/L in Gonghu Bay in 2008 [25]. Peak concentrations occurred in August [18, 20, 21] or October [20, 22, 25]. In this study, we observed a concentration of 44 *μ*g/L in Meiliang Bay, the highest among existing reports. We also found a high concentration (50 *μ*g/L) at Huzhou city on the south shore of Lake Taihu. There are two possible reasons why higher concentrations were observed in this study than in previous studies: microcystin concentrations may be increasing, possibly due to climate change, or concentrations at these locations have always been high and simply have not been documented until now. In either case, future surveys should be conducted more frequently and across a broader area to elucidate the overall condition of the lake. Long-term surveys should also be conducted to understand changes over time and to reveal the possible effects of climate change. 

### 4.2. Use of E/T Ratios to Characterize Algal Blooms

The characterization of algal blooms by their growth stage, using E/T ratios, is important for the formulation of remediation strategies. If a bloom is in the early stages of growth, nutrient control can suppress further growth and microcystin release. Conversely, if a bloom is in a later stage of growth, physical removal of algae from the lake is recommended. If a bloom is affected by other external factors, the control of these factors will be a means of bloom suppression. 

In the present study, one sample showed a high E/T ratio of 107%. This indicates that all microcystins were in the water rather than in algal cells. Therefore, the physical removal of algal cells and microcystin degradation treatment would be an effective measure against this bloom. Other samples showed lower E/T ratios, from 1.6% to 19%. These samples could be in the growth phase, and therefore nutrient control might be better countermeasure against these blooms. As there was a negative correlation between E/T ratio and NO_3_-N concentration and a positive correlation between E/T ratio and PO_4_-P, the suppression of phosphate concentration may also be an effective countermeasure in Lake Taihu. 

For a few lower E/T ratio samples (S02, M01, and M06), values ranged from 1.6% to 2.5%, although very large total cellular microcystin concentrations (35–50 *μ*g/L) were observed. If E/T ratio is related to growth condition, samples with lower E/T ratios would be expected to show relatively less growth and lower total cellular microcystin concentration. However, there seems to exist an apparent contradiction, which could be accounted for by the migration of algal cells. E/T ratio is primarily affected by growth conditions but can also be affected by external factors such as dilution or exchange with fresh media. In water, the wind-driven migration of an algal bloom could be the cause. This is because only algal cells would be moved by wind, while the surrounding water would not. Therefore, it is possible that samples with a high total cellular microcystin concentration and low E/T ratio were impacted by the migration of algal cells. In such situations, the removal of algal cells from windward areas would be useful for preventing further leeward hazards. 

### 4.3. Factors Affecting the Distribution of Microcystins

We investigated the relationships between the distribution of microcystins and other water quality parameters. Microcystins were correlated with chlorophyll-a and COD but showed only a weak correlation with DO and nutrients. Therefore, the observed algal bloom contained both live and dead cells, depending on sampling location and timing. E/T ratios were more strongly correlated with DO and nutrients than were microcystin concentrations. They also showed that one sample was in the death phase while the other samples were in the growth phase. Three samples showed disproportionately large total cellular microcystin concentrations with low E/T ratios. This could be attributed to the migration of algal cells, as described above, possibly driven by the southeast wind prevailing at Lake Taihu [19]. As shown in Figure 4, the belt of high-concentration sampling sites that cross the mouth of Meiliang Bay is perpendicular to wind direction, which supports the idea of southeast winds contributing to microcystin distribution. 

## 5. Conclusions

In this study, we investigated the occurrence and distribution of microcystins in Lake Taihu and reached the following conclusions.

- Microcystins occurred over large areas of Lake Taihu. There was spatial variation in the distribution of microcystins, and the highest concentrations were observed in southern parts of the lake.
- Microcystin concentration was well correlated with COD, chlorophyll-a, and turbidity. E/T ratios were better correlated with NO_3_-N, PO_4_-P, and DO than was microcystin concentration.
- The use of E/T ratio helped clarify the growth stage of algal blooms and highlighted the possibility of algal cell migration caused by prevailing southeast winds at Lake Taihu.
- Extensive monitoring is required to confirm the advantages of E/T ratios and to better understand the state of algal blooms across Lake Taihu.

## Supplementary Material

Detailed data are shown in the supporting material, Table S1.

## Figures and Tables

**Table 1:** Correlation coefficients for each parameter.

	COD	DOC	NO_3_-N	PO_4_-P	Temp.	DO	Turbidity	Chlorophyll-a	pH	ORP	EC
Extra cellular	0.75	0.37	0.01	0.24	−0.38	0.28	0.96	0.71	0.18	−0.20	−0.04
Total cellular	0.73	0.32	0.15	0.17	−0.31	0.18	0.98	0.61	0.13	−0.08	0.02

**Table 2:** Calculated E/T ratios.

Sample	Area	Date	Weather	Total MCs	Extra MCs	E/T ratio
				*μ*g/L	*μ*g/L	—
S02	Huzhou	29-Jul-10	Sunny	50	0.79	1.6%
M01	Meiliang Bay	30-Jul-10	Sunny	44	0.96	2.2%
M06	Meiliang Bay	2-Aug-10	Sunny	35	0.88	2.5%
M10	Meiliang Bay	2-Aug-10	Sunny	4.8	0.12	2.5%
G06	Gonghu Bay	1-Aug-10	Sunny	2.9	0.09	3.1%
G07	Gonghu Bay	1-Aug-10	Sunny	3.6	0.14	4.0%
M15	Meiliang Bay	2-Aug-10	Sunny	1.6	0.07	4.5%
G05	Gonghu Bay	1-Aug-10	Sunny	2.7	0.13	4.8%
M12	Meiliang Bay	2-Aug-10	Sunny	0.94	0.06	6.0%
M09	Meiliang Bay	2-Aug-10	Sunny	2.5	0.17	6.8%
G08	Gonghu Bay	1-Aug-10	Sunny	1.3	0.12	9.0%
M08	Meiliang Bay	2-Aug-10	Sunny	1.1	0.15	14.2%
M11	Meiliang Bay	2-Aug-10	Sunny	1.2	0.17	14.3%
M07	Meiliang Bay	2-Aug-10	Sunny	1.2	0.18	14.6%
M17	Meiliang Bay	2-Aug-10	Sunny	0.44	0.08	19.0%
G02	Gonghu Bay	30-Jul-10	Stormy	0.20	0.22	107%

**Table 3:** Correlation coefficients for each parameter with E/T ratios.

	COD	DOC	NO_3_-N	PO_4_-P	Temp.	DO	Turbidity	Chlorophyll-a	pH	ORP	EC
E/T ratio	−0.23	−0.02	−0.40	0.27	0.01	0.30	−0.33	0.27	0.31	−0.18	0.06
