# Supplementary material for: Occurrence and Distribution of Microcystins in Lake Taihu, China
Source: ScientificWorldJournal. 2013 Jun 16;2013:838176. doi: 10.1155/2013/838176 (PMC3703407; doi:10.1155/2013/838176)
Supplement: Supplementary file 1 — Detailed data are shown in the supporting material, Table S1. [file 838176.f1.docx]

**Supporting information**

Table S1. Sampling data.
